# Differential effects of the pharmacological stressor yohimbine on impulsive decision making and response inhibition

**DOI:** 10.1007/s00213-016-4337-3

**Published:** 2016-06-01

**Authors:** M. C. Schippers, D. Schetters, T. J. De Vries, T. Pattij

**Affiliations:** Department of Anatomy and Neurosciences, Neuroscience Campus Amsterdam, VU University Medical Center, De Boelelaan 1108, 1081 HZ Amsterdam, The Netherlands

**Keywords:** Impulsivity, Impulsive choice, Response inhibition, Delayed reward task, Stop-signal task, Noradrenaline, α2 noradrenergic receptor, Yohimbine, Stress

## Abstract

**Rationale:**

High levels of impulsivity have been associated with psychiatric disorders such as attention-deficit/hyperactivity disorder (ADHD) and substance abuse. In addition, acute stress is known to exacerbate many psychiatric symptoms in impulse control disorders.

**Objectives:**

The purpose of the current study was to investigate the acute effects of the pharmacological stressor yohimbine on response inhibition and impulsive choice.

**Methods:**

A group of male rats (*n* = 12) was trained in the delayed reward task (DRT) to assess impulsive choice. A separate group (*n* = 10) was trained in the stop-signal task (SST) to measure response inhibition. Upon stable responding, the effects of yohimbine (0, 1.25, 2.5, and 5 mg/kg i.p.) were tested in a Latin square design.

**Results:**

Acute yohimbine significantly increased the preference for the large and delayed reinforcer in the DRT, indicating a decrease in impulsive choice. On the contrary, the effect size of 1.25 mg/kg yohimbine on stop-signal reaction times correlated negatively with baseline performance, suggesting a baseline-dependent effect on response inhibition as measured in the SST.

**Conclusions:**

The current data suggest that the effects of the pharmacological stressor yohimbine on impulse control strongly depend on the type of impulsive behavior. Pharmacological stress decreased impulsive decision making, an observation that is in line with previously published rodent studies. By contrast, the lowest dose of yohimbine revealed a baseline-dependent effect on response inhibition. As such, the effects of yohimbine are largely comparable to the effects of psychostimulants on impulsivity and may support the notion of cross sensitization of stress and psychostimulants.

## Introduction

Maladaptive impulsive behavior is associated with many psychiatric disorders, such as drug addiction, obsessive-compulsive disorder, attention-deficit/hyperactivity disorder (ADHD), bipolar disorder, and pathological gambling (Fineberg et al. 2014; Moeller et al. 2001; Pattij and De Vries 2013). In addition, acute stress is known to exacerbate many psychiatric symptoms in impulse control disorders (Roberts et al. 2009). However, the role of acute stress in impulsive behavior has not been well characterized.

Impulsivity is a multifaceted concept (Evenden 1999), and it has been recognized that different forms of impulsive behavior can be dissociated on a neuroanatomical, neuropharmacological, and behavioral level (Evenden 1999; Pattij and Vanderschuren 2008). In this regard, two main forms of impulsive behavior have been recognized, namely, impulsive action and impulsive choice, which do not correlate on the individual level, suggesting distinct underlying neural mechanisms (Broos et al. 2012; Robinson et al. 2009; Solanto et al. 2001). Of these, the former can be defined as difficulties to inhibit either inappropriate or planned motor responses. The latter, impulsive choice is defined as the preference for a small immediate reward over a delayed but more beneficial reward. Since acute stress is possibly related to maladaptive impulsive behavior, the characterization of the effects of stress on different forms of impulsivity is of high importance, in order to provide a better understanding of the underlying neurobiological mechanisms and to eventually improve treatment opportunities.

Acute stress downregulates presynaptic α2 adrenergic receptors (Flugge et al. 2004), thereby increasing noradrenergic signaling (Abercrombie et al. 1988), which is suggested to be mimicked by the competitive α2 adrenoceptor antagonist yohimbine. This compound, yohimbine, is frequently used as a pharmacological agent to study acute stress effects, since the compound can be studied across species and provides the opportunity to titrate stress with varying doses. In humans, it has been shown that the administration of yohimbine leads to panic and anxious feelings, increased heart rate, and increased cortisol measures and noradrenaline metabolite levels (Bourin et al. 1998; Charney et al. 1984; Stine et al. 2002; Swann et al. 2005).

Several preclinical studies have shown that yohimbine increases impulsive action in the 5-choice serial reaction time task (5-CSRTT) in rats (Sun et al. 2010; Torregrossa et al. 2012) and the rat gambling task (Connolly et al. 2015), observations that are confirmed clinically in the continuous performance task (Swann et al. 2005; Swann et al. 2013). Recently, yohimbine has been reported to decrease impulsive choice in rats as measured in the delayed reward task (DRT) (Schwager et al. 2014), in contrast to the clinical observations on impulsive decision making which report increases by stress (Fields et al. 2014; Kimura et al. 2013; Lempert et al. 2012; Porcelli and Delgado 2009; Putman et al. 2010). Taken together, stress seems to increase some forms of impulsive behavior; however, this could not be confirmed in a preclinical DRT.

Despite the fact that yohimbine has been shown to impair measures of action impulsivity, this is the first study to explore its effects in the stop-signal task (SST). Although response inhibition is a form of impulsive action, it is conceptually different from impulsive action as measured in the 5-CSRTT. In the SST, a task which assesses response inhibition, estimated stop-signal reaction times are an indication of action cancellation (Verbruggen and Logan 2008), whereas premature responses in the 5-CSRTT reflect the inability to inhibit prepotent actions (Robbins 2002).

The purpose of the current study was to investigate the effects of the pharmacological stressor yohimbine on different forms of impulsive behavior. More specifically, we examined the effect of yohimbine on response inhibition and impulsive choice in two translational rodent paradigms. To this end, impulsive choice was measured using the DRT, a task in which rats are allowed to choose a small immediate or large delayed reward. The SST was used to measure response inhibition. Upon stable baseline performance in the two tasks, the effects of yohimbine on measures of impulsive behavior were tested. Based on previous preclinical findings with acute yohimbine on different impulsivity tasks (Connolly et al. 2015; Schwager et al. 2014; Sun et al. 2010; Torregrossa et al. 2012), we hypothesized that the administration of yohimbine will decrease impulsive choice and will increase response inhibition.

## Methods

### Animals

Twenty-four male Wistar rats (Harlan, Horst, the Netherlands) weighing 250–275 g at the start of the experiment served as the subjects and were run in two separate experimental groups. The rats were housed in pairs in enriched Makrolon cages and kept under standard housing conditions under a reversed 12-h light/dark schedule (lights on at 19:00 h). For the purpose of the behavioral tasks, the animals were food restricted and maintained at 90 % of their free-feeding body weight. Water was available ad libitum. All experiments were approved by the Animal Ethical Committee of the VU University and VU University Medical Center of Amsterdam.

### Behavioral tasks

#### Apparatus

Both tasks were conducted in 12 identical operant chambers (Med Associates Inc., St. Albans, USA) which were housed in sound-attenuating ventilated cubicles. One wall contained an array of five nose poke holes that could be illuminated and had an infrared beam for nose poke detection. On the opposite wall, a food magazine was situated, where the reward (45-mg precision pellets; BioServ, Frenchtown, USA) was delivered. A white house light was situated on the same wall as the food tray.

### Delayed reward task

A detailed description of the delayed reward paradigm as employed in our laboratory has been described previously (van Gaalen et al. 2006b). For the purpose of the DRT, nose poke holes 2, 3, and 4 in the operant chambers were used. After habituating the rats to the operant chambers and food pellets, the animals were trained to make a nose poke in one of the three holes, which resulted in a delivery of a food pellet. Next, the animals were required to first make a response in the central unit, followed by a response in either the left or right unit, which resulted in a delivery of a food pellet. In the following stages of training, each session was divided into 5 blocks of 12 trials, each block starting with 2 forced trials, during which, after initiating the trial through a nose poke into the central unit, either the left unit or the right unit was illuminated in a counterbalanced fashion. In the next 10 trials, the animals had a free choice and both the left and right units were illuminated. Poking into one position resulted in the immediate delivery of a small reinforcer (one food pellet), whereas a nose poke into the other position resulted in the delivery of a large, but delayed, reinforcer (four food pellets). Over sessions, the within-session delays for the large reinforce were increased to 0, 5, 10, 20, and 40 s per block. If an animal did not make a response during this choice phase within 10 s, an intertrial interval (ITI) was initiated and the trial was counted as an omission. The position associated with the small and large reinforcer was always the same for each individual and counterbalanced for the group of rats. Responding into non-illuminated units during the test was recorded but had no further programmed consequences. The behavioral measure to assess task performance, i.e., the percentage preference for the large reinforcer as a function of delay, was calculated per delay block of 10 trials within a session as the number of choices for the large reinforcer choices/(number choices large + small reinforcers) × 100. In addition, the total number of omitted started trials and choice trials per block of 10 trials within a session and the average response latencies to start a trial and to make a response in the nose poke hole associated with the small and large rewards after onset of the stimulus light in the corresponding hole were calculated. Furthermore, hyperbolic curves for the percentage preference were fitted on the individual data by the equation *V* = *A*/(1 + *kD*), where *V* is the preference for the large reward after a delay of *D* in seconds, *A* is the preference for the large reward at *D* = 0 s, and *k* describes the steepness of the discounting curve (Mazur 2006). Based on the estimated hyperbolic curve, the indifference point, the delay for which the rats switched their preference over to the immediate, small reward (i.e., the delay on which the preference for large reward <50 %) was calculated.

### Stop-signal task

#### Shaping

For the purpose of the stop-signal task, only nose poke hole 3 and one of the outer right or left holes were used (counterbalanced for all subjects). The stop-signal task as employed in our laboratory has been described more elaborately elsewhere (Pattij et al. 2009). Briefly, during initial shaping for two consecutive sessions, both the middle nose poke hole and the outer holes to the right or left were illuminated. A nose poke into either one of the two active holes extinguished the visual stimuli in both holes and resulted in delivery of a pellet. After an ITI of 30 s, the next trial started. Nose poking within this ITI period did not have any programmed consequences. A session ended after 30 min or 100 trials, whichever occurred first.

#### Shaping: go trials

During the next phase, only the stimulus light in the middle nose poke hole was illuminated (start stimulus). A response into the active middle hole switched off the stimulus light and was followed by the illumination of the stimulus light (go stimulus) in the outer left or right hole. A nose poke into the illuminated hole switched off the stimulus light and resulted in the delivery of a pellet. After an ITI of 5 s, the next trial started. Responding in the start stimulus hole during the presentation of the go stimulus was counted as perseverative start pokes, whereas prestimulus responses into the go stimulus hole resulted in a time-out period of 5 s. Subsequently, the response requirements into the start stimulus hole before the onset of a go stimulus were varied into a variable ratio 2 schedule (VR2, i.e., either FR1, FR2, or FR3) to avoid the development of a prepotent response pattern from the start stimulus to the go stimulus hole and to ensure that the animals waited until the appearance of a go stimulus. During this phase, the rats were trained until they reliably completed 100 successful go trials. Following this phase, a limited hold period was introduced for the go stimulus and only during this period was the go stimulus present. Initially, the limited hold was set at 5 s, and in subsequent sessions, was individually titrated to meet performance criterion of 80 % successful hits and <20 % prestimulus responses. Omissions of a go stimulus response within the limited hold resulted in a 5-s time-out period, during which both the house light and stimulus light were turned off.

#### Shaping: introduction stop signal

During the final training phase, a stop signal was introduced in 25 % of all trials. Initially, this stop signal (duration 50 ms, frequency 4500 Hz, and intensity 80 dB) was contingent with the appearance of the go signal. Responding during the onset of the stop signal or during the limited hold immediately extinguished the go stimulus and house light, turned off the stop signal, and was followed by a 5-s time-out. In contrast, if the animal successfully refrained from responding during a stop trial, a pellet was delivered. Initially, the limited hold during stop and go trials were equal; however, when performance during stop trials was below 80 % successfully inhibited stop trials, the limited hold during stop trials was lowered over sessions in steps of 50–100 ms until animals improved performance. Subsequently, the limited hold was then gradually increased in these individuals over sessions until the limited hold during both the go and stop trials were equal. As soon as animals reached the criterion of approximately 90 % successfully inhibited stop trials, delays for the onset of the stop signal were introduced. The stop-signal delays (SSDs) were presented in a pseudorandom order, and to compensate for differences between rats, SSDs were based on each individual rat’s mean reaction time on go trials in the preceding drug-free training session. SSDs were calculated as follows: mean go reaction time (mean GoRT) minus either 50, 75, 150, 300, and 500 ms. In addition, an equal amount of zero delays were presented during sessions. Drug testing commenced upon stable baseline performance for at least five consecutive sessions, i.e., 80 % accuracy during go trials and a significant SSD-dependent decrease in correctly inhibited stop trials.

#### Stop-signal paradigm: estimation stop-signal reaction time and correction for omissions during go trials

Stop-signal reaction times (SSRTs) were estimated with the integration method, which is less influenced by skewness of the reaction time distribution (Verbruggen et al. 2013). The performance of the rats was analyzed according to the assumptions of the race model, which assumes that go and stop processes are independent from each other (Logan and Cowan 1984).

### Drugs

Yohimbine hydrochloride (Sigma, St. Louis, MO, USA) was dissolved in distilled water. On test days, yohimbine was freshly prepared and intraperitoneally injected in a volume of 1-ml/kg body weight according to a Latin square within-subject design. Doses (1.25, 2.5, and 5 mg/kg) were based on previous studies investigating the effects of acute yohimbine administration on impulsive behavior (Schwager et al. 2014; Sun et al. 2010; Torregrossa et al. 2012). Upon stable baseline responding and prior to pharmacological challenges, the rats were habituated to the injection procedures to rule out possible stressful effects of the injection. In all experiments, vehicle or yohimbine was administered 30 min before testing on Tuesdays and Fridays with baseline training sessions on the other weekdays.

### Statistical analyses

All data are presented as mean ± standard errors of the mean. Data were analyzed with repeated measures analysis of variance (ANOVA) with drug dose, delay for the large reward (DRT), and SSD (SST) as within-subject variables using IBM SPSS Statistics version 20 (IBM, New York, USA). In case of violation of homogeneity, Mauchly’s test for equal variances corrected degrees of freedom and resulting more conservative *p* values were used for subsequent analyses. In case of a statistical significant main effect, further post hoc testing was conducted using pairwise comparisons with Bonferroni adjustments for multiple comparisons. Correlation analyses were performed using Pearson’s correlation. The level of statistical significance was set at *p* < 0.05.

## Results

### Effects of yohimbine on impulsive decision making

**Fig. 1:**
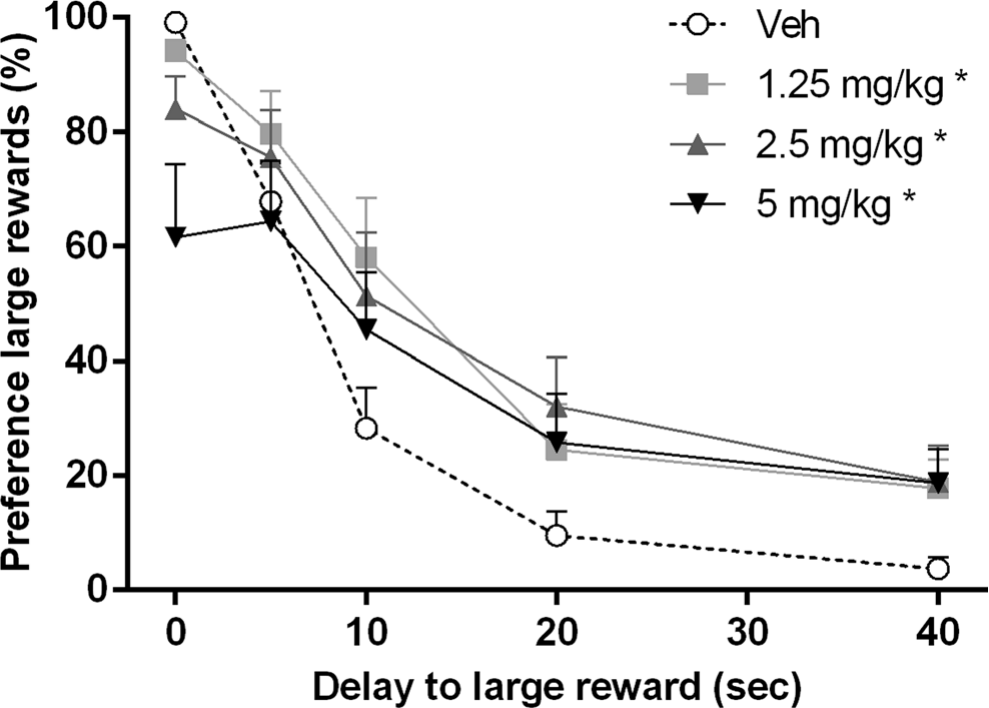
Effects of yohimbine (0, 1.25, 2.5, and 5 mg/kg i.p.) on the mean percentage preference for the large reward as measured in the DRT (*n* = 12). *p < 0.05 vs vehicle condition

**Fig. 2:**
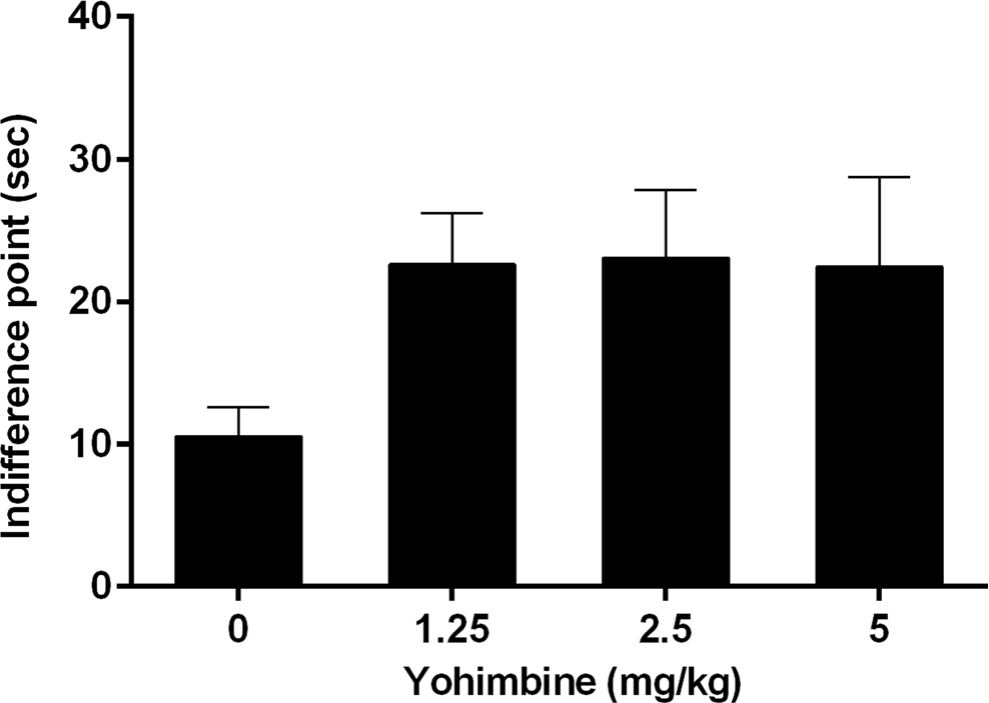
Effects of yohimbine (0, 1.25, 2.5, and 5 mg/kg i.p.) on the mean indifference point as measured in the DRT (*n* = 12)

In the DRT, the performance after vehicle administration did not differ significantly from the last 3 days of baseline training [preference for large reward day *F*(3,33) = 0.23, N.S.; days × delay *F*(12,132) = 0.98, N.S.; start trial omissions *F*(3,33) = 1.44, N.S.; choice trial omissions *F*(3,33) = 0.34, *ε* = 0.65, N.S.; forced trial omissions *F*(3,33) = 0.67, N.S.] (Table 1). Increasing the delays for the large reward significantly decreased the mean percentage preference for the large reward (delay *F*(4,44) = 93.68, *p* < 0.001; Fig. 1). However, the main effect of yohimbine on impulsive decision making did not reach significance [dose *F*(3,33) = 2.33, *p* = 0.092]; there was a strong significant interaction between the dose of yohimbine and the delay for the large reward [dose × delay *F*(12,132) = 3.45, *p* < 0.001]. This indicates that across doses, yohimbine differentially affected impulsive decision making. Further post hoc pairwise comparisons revealed that all three doses of yohimbine significantly increased preference for the large reward compared to the vehicle condition (all *p*s < 0.05). In contrast, this decreased preference for the large reward was not reflected by significant changes in indifference points (*F*(3,30) = 2.41, *p* = 0.087; Fig. 2). Notably, when the highest dose of 5 mg/kg yohimbine was administered, 5 out of 12 rats showed <50 % preference for the large reward during the 0-s delay trials. The response latencies to start a trial were significantly altered by yohimbine administration [*F*(3,33) = 12.60, *p* < 0.001], and further post hoc analyses revealed a significant increased latency by the highest dose of 5 mg/kg compared to all other doses (*p* < 0.05; Table 2). Further in-depth analyses by excluding all animals from the 5-mg/kg yohimbine dose due to decreased preference for large rewards at 0-s delay and increased response latency did reveal a strongly significant increase in indifference point [*F*(2, 22) = 7.14, *p* = 0.004]. Additional post hoc pairwise comparisons showed that 1.25 and 2.5 mg/kg yohimbine significantly increased the indifference point compared to the vehicle condition (*p* < 0.05).

**Table 1:** Baseline parameters of the last three training days before start of the experiments as measured in the DRT (*n* = 12)

		Baseline		
		Day 1	Day 2	Day 3
Preference large reward (%)	0 s	85.8 ± 8.6	93.2 ± 3.6	94.5 ± 2.1
	5 s	74.2 ± 8.6	67.6 ± 8.9	77.3 ± 8.2
	10 s	23.2 ± 7.6	31.6 ± 7.2	27.8 ± 8.3
	20 s	7.7 ± 2.5	6.9 ± 1.9	5.8 ± 1.9
	40 s	4.4 ± 2.0	3.5 ± 2.4	2.5 ± 1.3
Omission start (average no. per session)		0.6 ± 0.2	0.6 ± 0.1	0.4 ± 0.1
Omission choice (average no. per session)		0.6 ± 0.1	0.7 ± 0.2	0.6 ± 0.1
Omission forced trials (average no. per session)		0.4 ± 0.1	0.3 ± 0.1	0.4 ± 0.1

**Table 2:** Effects of yohimbine (0, 1.25, 2.5, and 5 mg/kg i.p.) on the average number of ITI responses, omissions to start a trial, omissions during the choice phase, omissions during the forced trials, and response latencies for a small reward and large reward and to start a trial as measured in the DRT (*n* = 12)

	Yohimbine (mg/kg)			
	0	1.25	2.5	5
ITI pokes (average no. per session)	111.2 ± 38.1	175.1 ± 65.0	183.8 ± 92.9	142.7 ± 72.8
Omission start (average no. per session)	0.6 ± 0.2	0.5 ± 0.1	0.8 ± 0.1	1.1 ± 0.3
Omission choice (average no. per session)	0.7 ± 0.2	0.7 ± 0.2	0.8 ± 0.1	1.3 ± 0.4
Omission forced trials (average no. per session)	0.3 ± 0.1	0.5 ± 0.1	0.5 ± 0.1	0.9 ± 0.1
Latency small reward (s)	0.62 ± 0.1	0.66 ± 0.1	1.08 ± 0.2	1.07 ± 0.2
Latency large reward (s)	0.71 ± 0.1	0.70 ± 0.1	0.74 ± 0.1	1.04 ± 0.2
Latency start trial (s)	1.86 ± 0.1	1.55 ± 0.1	1.79 ± 0.2	2.56 ± 0.2*

*p < 0.05 compared to 0, 1.25, and 2.5 mg/kg

In order to explore whether yohimbine had differential effects on the indifference point depending on baseline performance under vehicle conditions, correlation analyses were performed. Neither the effect size of yohimbine on the indifference point, as averaged over the three drug doses, correlated significantly with vehicle performance [Pearson’s *r* = −0.02, N.S.] nor the effect size of each dose of yohimbine separately revealed a significant correlation with vehicle performance [1.25 mg/kg, Pearson’s *r* = −0.23, N.S.; 2.5 mg/kg, Pearson’s *r* = −0.41, N.S.; 5 mg/kg Pearson’s *r* = −0.14, N.S.] (data not shown).

Other task parameters, such as the number of ITI responses [*F*(3, 33) = 1.79, *ε* = 0.42, N.S.], omitted starts of a trial [*F*(3, 33) = 2.09, *ε* = 0.85, N.S.], omitted choice trials [*F*(3, 33) = 1.71, *ε* = 0.71, N.S.], or omitted forced trials [*F*(3, 33) = 1.09, N.S.], were not significantly altered by yohimbine (Table 2).

### Effects of yohimbine on response inhibition

Two out of 12 rats were excluded from all analyses based on their performance in the SST. One rat did not show stable performance during test days as indicated by a low and highly variable number of started trials (varying from 16 to 94 % of the average started trials by the group). A second rat did not satisfy the race model on two test days (vehicle and 1.25 mg/kg yohimbine).

The estimated SSRT, the main measure for response inhibition, did not differ significantly from the last 3 days of baseline training and performance after vehicle administration [*F*(3,27) = 1.74, *ε* = 0.65, N.S.]. In addition, the percentage of correctly inhibited responses [days *F*(3,27) = 0.82, N.S.; days × delays *F*(15,135) = 1.65, N.S.] and the percentage of omitted go trials [*F*(3,27) = 1.81, N.S.] were not significantly different between the baseline and vehicle performance. The mean GoRT was however significantly increased after vehicle administration compared to baseline training days [*F*(3,27) = 9.90, *p* < 0.001]. The post hoc analyses showed that vehicle administration significantly increased the mean GoRT compared to two of three baseline days (vehicle 497.0 ± 34.0 ms, baseline day 1 470.8 ± 36.2 ms, baseline day 2 466.6 ± 34.2 ms, *p* < 0.05; Table 3).

**Table 3:** Baseline parameters of the last three training days before start of the experiments as measured in the stop-signal task (*n* = 12)

		Baseline		
		Day 1	Day 2	Day 3
Estimated SSRT (ms)		0.2 ± 0.0	0.2 ± 0.0	0.2 ± 0.0
Correct inhibition (%)	0 ms	95.9 ± 2.3	100 ± 0	97.0 ± 1.5
	500 ms	97.5 ± 1.7	100 ± 0	96.3 ± 1.9
	300 ms	86.3 ± 4.7	85.9 ± 4.4	92.5 ± 3.3
	150 ms	57.0 ± 5.0	53.8 ± 5.4	42.3 ± 6.8
	75 ms	33.6 ± 5.3	25.7 ± 5.7	27.9 ± 3.6
	50 ms	27.7 ± 3.1	34.3 ± 8.3	37.9 ± 5.5
Omitted go trials (%)		15.8 ± 1.8	18.6 ± 1.4	18.7 ± 1.7
Mean GoRT (ms)		475.5 ± 35.3	470.8 ± 36.2	466.6 ± 34.2

**Fig. 3:**
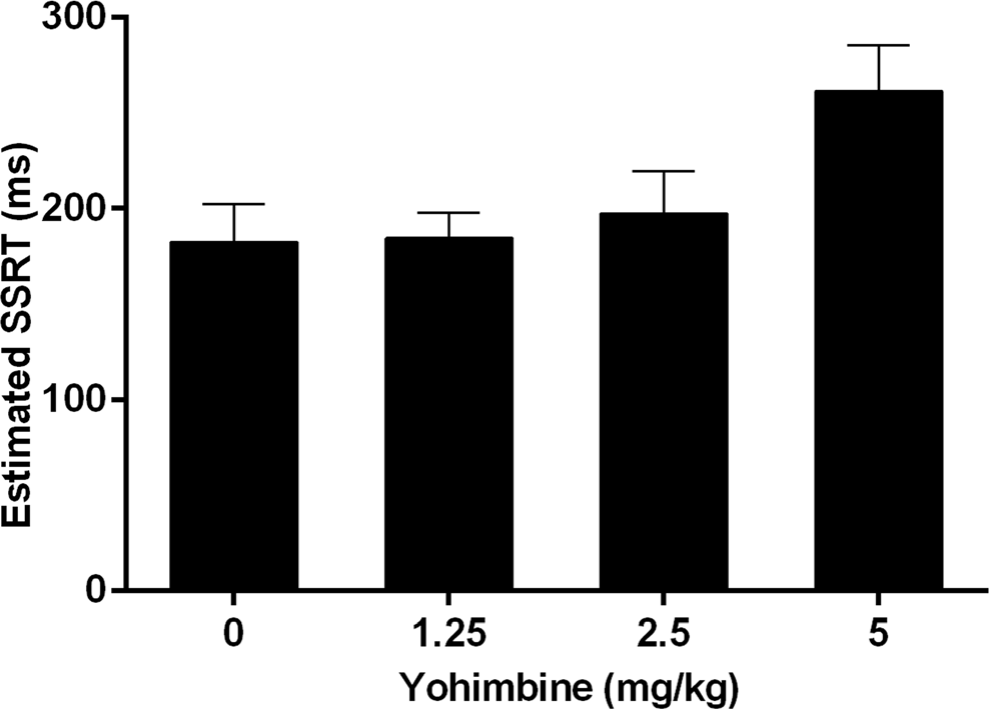
Effects of yohimbine (0, 1.25, 2.5, and 5 mg/kg i.p.) on the estimated stop-signal reaction time as measured in the SST (*n* = 10)

**Fig. 4:**
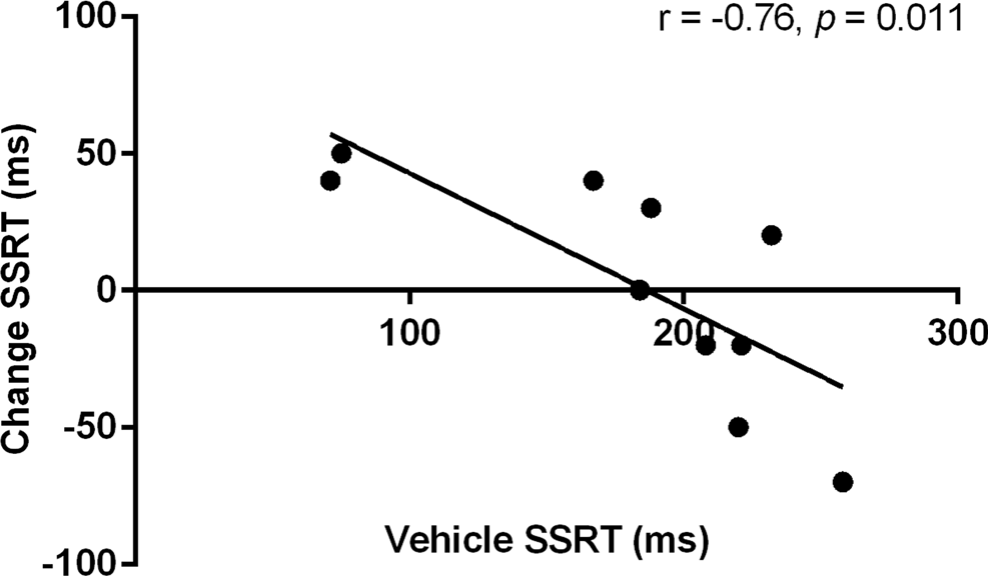
Effect size as a correlation between the change in SSRT for 1.25 mg/kg yohimbine and baseline SSRT as measured in the SST (*n* = 10)

The estimated SSRT was significantly altered by yohimbine (*F*(3, 27) = 5.06, *ε* = 0.47, *p* = 0.034; Fig. 3). However, further post hoc analyses revealed no significant differences between the various doses of yohimbine. In order to explore whether yohimbine had differential effects on the estimated SSRT depending on baseline performance under vehicle conditions, a correlation analysis was performed. The analyses of the effect size of each dose of yohimbine separately revealed a significant negative correlation for 1.25-mg/kg dose of yohimbine (Pearson’s *r* = −0.76, *p* = 0.011; Fig. 4), whereas the other doses did not significantly correlate with vehicle SSRT (2.5 mg/kg, Pearson’s *r* = 0.14, N.S.; 5 mg/kg, Pearson’s *r* = 0.60, N.S.).

**Fig. 5:**
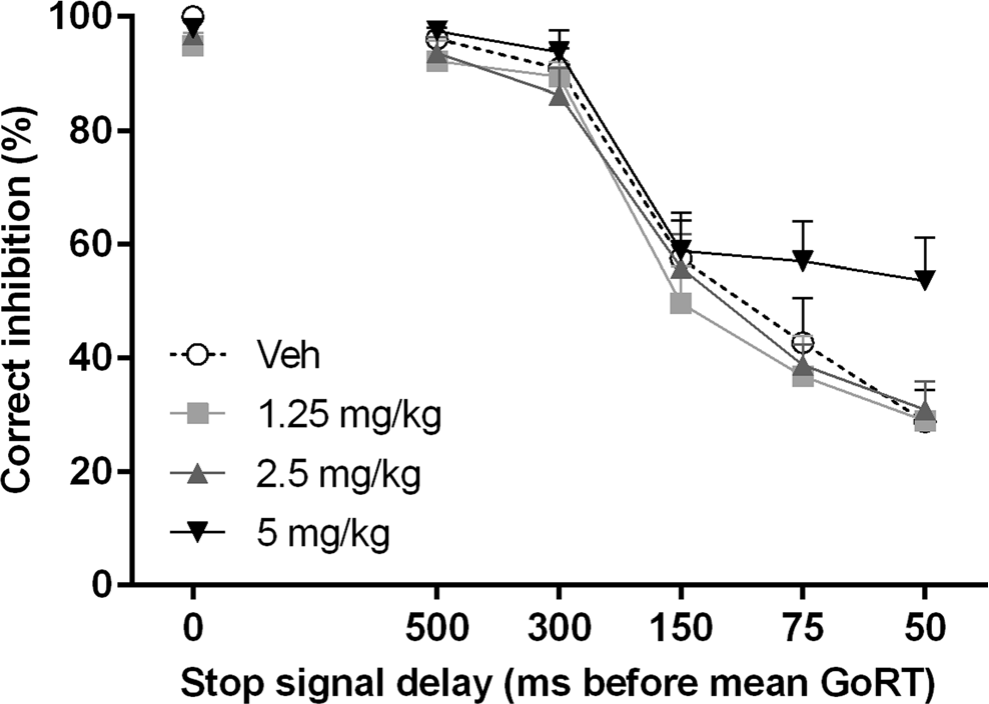
Effects of yohimbine (0, 1.25, 2.5, and 5 mg/kg i.p.) on the mean percentage of correctly inhibited stop trials with varying stop-signal delays before the mean GoRT as measured in the SST (*n* = 10)

**Fig. 6:**
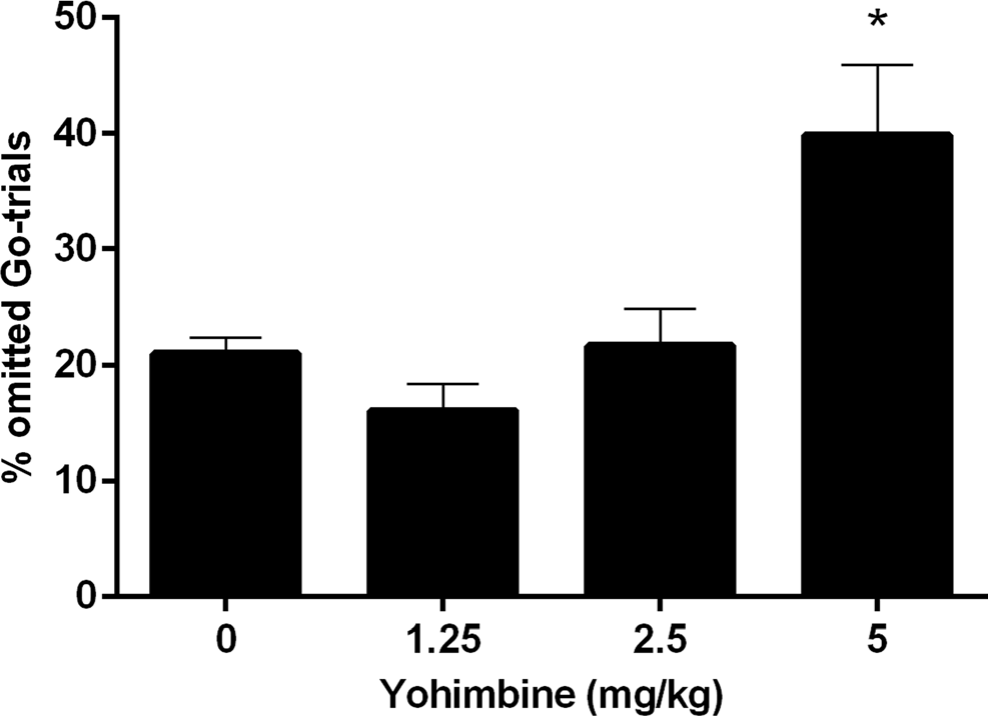
Effects of yohimbine (0, 1.25, 2.5, and 5 mg/kg i.p.) on the percentage of omitted go trials as measured in the SST (*n* = 10). The number of omitted go trials is expressed as the percentage of total go trials. *p < 0.05 compared to 1.25 and 2.5 mg/kg

**Fig. 7:**
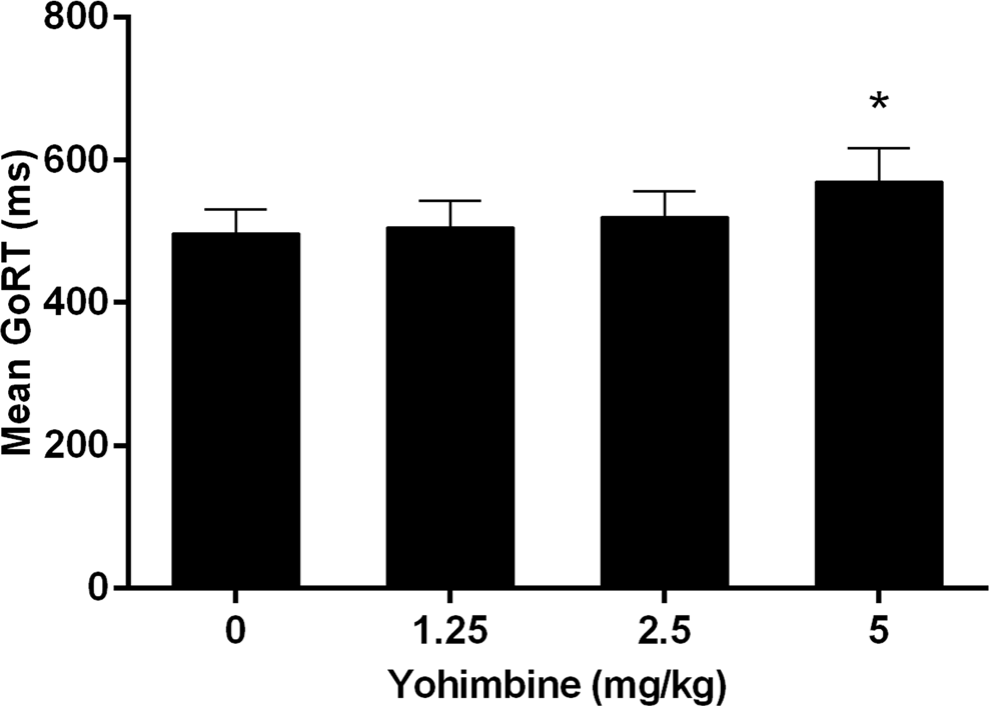
Effects of yohimbine (0, 1.25, 2.5, and 5 mg/kg i.p.) on the mean go reaction time as measured in the SST (*n* = 10). *p < 0.05 compared to 0 and 1.25 mg/kg

In addition to its effects on the estimated SSRT, yohimbine significantly increased the percentage of correctly inhibited responses (drug *F*(3,33) = 3.42, *p* = 0.028; Fig. 5). Further post hoc analyses revealed no significant differences between the various doses of yohimbine. Moreover, the number of omitted go trials was significantly increased [*F*(3,27) = 10.27, *ε* = 0.70, *p* = 0.001]. The post hoc comparisons revealed that the highest dose of 5 mg/kg yohimbine significantly increased the number of omitted go trials compared to 1.25 (*p* = 0.008) and 2.5 mg/kg yohimbine (*p* = 0.028) and thus reduced the number of successful hits (go accuracy) compared to these doses (Fig. 6). In addition, the mean GoRT was significantly increased by the highest dose of yohimbine (*F*(3,27) = 8.97, *ε* = 0.54, *p* = 0.004; post hoc pairwise comparisons 0 vs 5 mg/kg, *p* = 0.021; 1.25 vs 5 mg/kg, *p* = 0.014; Fig. 7).

## Discussion

The current study investigated the effects of acute challenges with the pharmacological stressor yohimbine on two distinct forms of impulsive behavior, namely, impulsive choice and response inhibition. Data indicate that yohimbine increased the preference for the large delayed reward in the DRT, indicating that pharmacologically induced stress attenuated impulsive choice by increasing self-controlled choice. By contrast, the measures of response inhibition capacities in the SST showed a baseline-dependent effect of the lowest dose of yohimbine on the estimated stop-signal reaction times. This suggests that pharmacologically induced stress improves response inhibition in high-impulsive individuals, whereas response inhibition is decreased in low-impulsive individuals.

In the present study, acute yohimbine administration increased the preference for the large reward. Parameters measuring aspects of general performance or motivation, such as the number of omitted trials and response latencies, were not affected, suggesting that there is a specific effect of yohimbine on decision making as measured in the DRT. The observation that acute yohimbine decreased impulsive choice is in line with a recent observation. Using a similar experimental design, Schwager and co-workers (2014) showed decrements in impulsive choice upon yohimbine challenges, using highly palatable soy emulsion as a reward.

It is hypothesized that acute stress can induce habitual responding (Schwabe and Wolf 2009). This notion would fit with previous findings in the DRT indicating that acute yohimbine biases choice for the small reward in a descending delay version of the task (Schwager et al. 2014), i.e., promotes perseveration for initial action selection as a result of habitual responding. The present study, however, did not employ a descending delay version of the DRT. In addition, the different SSDs in the SST were presented in a random order, preventing the interpretation of habit formation in this latter task. Therefore, in the present study, effects of habitual responding, altered reward sensitivity of aversion to delays by yohimbine cannot be ruled out.

Notably, in the DRT, the highest yohimbine dose of 5 mg/kg revealed decreased preference for the large reward at 0-s delay trial and, furthermore, increased response latencies to start a trial, which is likely a non-specific effect of this dose on behavioral performance in the task. Although it remains speculative, this suggests that high doses of yohimbine have sedative effects or decrease reward sensitivity. In line with this, increased omission rates at a comparable 5 mg/kg yohimbine dose have also been reported in the 5-CSRTT (Sun et al. 2010; Torregrossa et al. 2012). Furthermore, it has been suggested that yohimbine doses of 5 mg/kg and higher exert anxiogenic effects (Cole et al. 1995; Singewald et al. 2003; Sun et al. 2010). In addition, others and the current study show effects on impulsive behavior with doses lower than 5 mg/kg (Connolly et al. 2015; Schwager et al. 2014; Sun et al. 2010; Torregrossa et al. 2012), suggesting that effects of yohimbine on impulsivity and anxiety are dissociable. Although we did not assess anxiety-related behavior, our observation that doses lower than 5 mg/kg did not alter measures of general motivation or locomotor activity indicates that these doses most likely alter cognitive processes subserving impulsivity, rather than emotionally driven effects.

Previous work has shown that systemic administration of yohimbine can induce c-Fos activity in the prefrontal cortex (PFC) (Singewald et al. 2003). In addition, yohimbine-evoked increases in impulsive action in 5-CSRTT have been associated with increased CREB in the orbitofrontal cortex (OFC) (Sun et al. 2010). Both the PFC and OFC have been shown to play a crucial and differential role in impulsive action measured in the 5-CSRTT and impulsive decision making (Cardinal 2006; Pattij and Vanderschuren 2008; Winstanley 2011). For example, medial PFC lesions did not affect premature responding in the 5-CSRTT (Muir et al. 1996), whereas medial PFC lesions decreased the preference for the large reward at the 0-s delay and displayed less delay discounting compared to controls (Cardinal et al. 2001). Likewise, OFC lesions were found to decrease impulsive choice (Mar et al. 2011; Winstanley et al. 2004) but were found to increase premature responding in the 5-CSRTT (Chudasama et al. 2003). The apparent differential involvement of these brain regions in impulsive action and impulsive choice is in line with the opposite effects of yohimbine in the 5-CSRTT (Sun et al. 2010; Torregrossa et al. 2012) and the DRT. Although it remains speculative, this may indicate that the effects of acute yohimbine on prefrontal and orbitofrontal cortex functioning might in part explain the current findings.

Interestingly, in contrast to the findings with yohimbine, there is limited available evidence of effects of acute non-pharmacological stress on impulsive decision making. For example, restraint stress in rats has been shown to decrease preference for a more costly reward in effort-based decision making, but this same stress protocol had no effect on delay discounting (Shafiei et al. 2012). Furthermore, in the same study, corticosterone administration had no effect on decision making. Explanations for the discrepancy between the effects of pharmacological and non-pharmacological stress on impulsive behavior might relate to differences between types of stressors. Although we did not employ this, non-pharmacological stress can be induced for instance by exposure to pain-related stimuli or social stress that might affect behavior differently (Armario et al. 1991).

To our knowledge, the current study is the first to investigate the effects of yohimbine on response inhibition in the SST. The overall analysis of the SST results suggests that yohimbine decreased aspects of response inhibition, as measured by increased estimated stop-signal reaction times. Notably, similar to the DRT, the highest dose of 5 mg/kg yohimbine induced non-specific behavioral effects in the SST, by increasing omission rates and increasing reaction times for the go trials. Thus, this would argue against strong effects of yohimbine-induced stress on response inhibition per se. However, since many studies in the SST have shown baseline-dependent pharmacological effects (de Wit et al. 2002; Eagle et al. 2007; Pattij et al. 2009), further in-depth correlation analyses indeed revealed a baseline-dependent effect of yohimbine on response inhibition. These analyses showed that a low dose of yohimbine improved response inhibition in individuals with high-baseline SSRTs but impaired response inhibition in individuals with low-baseline SSRTs. Thus, this may suggest that acute stress can exacerbate symptoms of impaired impulse control. Although currently limited to findings mainly in the 5-CSRTT, it has been shown that trait impulsivity is related to differences in neurobiological makeup (Jupp et al. 2013). These effects were specific for the lowest dose of 1.25 mg/kg yohimbine and were not prevalent in the other doses. In contrast, no baseline-dependent effects of yohimbine were found on impulsive choice, suggesting that acute stress, induced by a low dose of yohimbine, appears to specifically alter response inhibition in a baseline-dependent manner.

Response inhibition and delay discounting represent different forms of impulsive behavior. The present study corroborates the findings that both types of behavior do not seem to correlate in the majority of studies (Broos et al. 2012; Robinson et al. 2009; Solanto et al. 2001). Impaired action inhibition, as measured by increased premature responses in the 5-CSRTT, is yet another form of impulsive behavior (Robbins 2002). Action inhibition and action cancellation are considered separate forms of impulsive action. Action inhibition as measured in the 5-CSRTT has been shown to be increased upon yohimbine administration (Sun et al. 2010; Torregrossa et al. 2012). The current differential effects of yohimbine on action cancellation and action inhibition further support dissociations between these measures of impulsivity, in addition to the dissociation between impulsive action and impulsive choice.

The noradrenergic system has been implicated to play an important role in the modulation of stress. Acute stress has been shown to reduce presynaptic α2 adrenoceptors (Flugge et al. 2004), thereby increasing noradrenergic signaling (Abercrombie et al. 1988), which is suggested to be mimicked by yohimbine. Previous studies have shown that the reinstatement of food and alcohol seeking resulting from comparable concentrations of yohimbine as used in the current study can be reversed by antagonizing the corticotrophin-releasing factor receptor, which plays an important role in the stress response (Ghitza et al. 2006; Marinelli et al. 2007). Besides the modulation of stress, noradrenaline is also implicated in impulsive behavior (Baarendse and Vanderschuren 2012; Bari et al. 2009; Pattij and Vanderschuren 2008; Pattij et al. 2012; Robinson et al. 2007; Winstanley 2011). Specifically, the beneficial effect of noradrenergic reuptake inhibitors in reducing impulsivity-related symptoms in ADHD has highlighted the importance of noradrenaline as an important modulator of impulsive behavior. Moreover, noradrenaline has also been implicated in other cognitive processes, including working memory (Arnsten and Jin 2014; Chamberlain et al. 2006) and behavioral flexibility (Lapiz and Morilak 2006).

Interestingly, the current and earlier reported effects of the stressor yohimbine seem comparable to the effects of psychostimulants on impulsive behavior. For instance, impulsive choice is attenuated by acute administration of the psychostimulants cocaine (Winstanley et al. 2007), methylphenidate (van Gaalen et al. 2006b), and amphetamine (Baarendse and Vanderschuren 2012; van Gaalen et al. 2006b; Winstanley et al. 2005), although some studies have shown contradicting results in this respect (Cardinal et al. 2000; Evenden and Ryan 1996; Stanis et al. 2008). Furthermore, amphetamine and methylphenidate improve response inhibition in the SST only in rats with a poor baseline performance (Eagle et al. 2007; Feola et al. 2000), similar to the present observations with yohimbine in the SST. Strikingly, amphetamine (Baarendse and Vanderschuren 2012; Cole and Robbins 1989; Pattij et al. 2007; van Gaalen et al. 2006a; van Gaalen et al. 2009), methylphenidate (Milstein et al. 2010; Pattij et al. 2012), cocaine (van Gaalen et al. 2006a; Winstanley et al. 2007), and nicotine (van Gaalen et al. 2006a) increase impulsive action as measured in the 5-CSRTT comparable to the acute effects of yohimbine (Sun et al. 2010; Torregrossa et al. 2012).

The remarkable similarity between yohimbine and psychostimulants can be explained by the fact that psychostimulants have been shown to target both dopaminergic and noradrenergic signaling (Florin et al. 1994; Kuczenski and Segal 1997; McKittrick and Abercrombie 2007; Ritz and Kuhar 1989), which is suggested to contribute to their effects on impulsive behavior (Pattij and Vanderschuren 2008). Similarly, yohimbine not only elevates noradrenaline signaling but also increases dopamine release (Tanda et al. 1996). This is in line with the observation that stress leads to increased striatal dopaminergic signaling (Sorg and Kalivas 1991; Wang et al. 2005), resulting in sensitization of dopaminergic motivation systems, which are involved in impulsive behavior and other psychiatric disorders related to stress such as substance abuse. This in turn may contribute to cross sensitization of stress and psychostimulants or other drugs of abuse, which is hypothesized to underlie the relation between stress and increased risk of substance abuse and relapse (Covington and Miczek 2001; de Jong et al. 2005; Lijffijt et al. 2014).

A limitation of the current study is that the behavioral effects of yohimbine may stem from actions of yohimbine at non-adrenergic receptors such as dopamine and serotonin (Newman-Tancredi et al. 1998; Tanda et al. 1996). Therefore, the biological mechanisms underlying the behavioral effects observed here remain speculative. Future research is needed to determine which neurotransmitter systems specifically are involved in the behavioral effects of yohimbine on impulsivity. A second possible limitation of this study is that the effects of yohimbine may be partly attributed to the possible sedative or motivational effects reflected by the increased omissions and response latencies observed with the highest dose of yohimbine. Also, as mentioned earlier, yohimbine might influence reward sensitivity or delay aversion, since the highest dose of yohimbine resulted in decreased preference for the large reward at 0-s delay in the DRT. Further research is warranted to examine whether these different aspects are at play in the effects of yohimbine.

In conclusion, acute systemic administration of the pharmacological stressor yohimbine decreased impulsive decision making in the DRT. By contrast, yohimbine exerted baseline-dependent effects on measures of response inhibition in the SST. Therefore, the current findings suggest differential involvement of stress in impulsive behavior. Since stress is an important vulnerability factor for various psychiatric disorders with comorbid maladaptive impulsivity, the current findings are valuable and provide further evidence for the multifaceted nature of impulsivity and its modulation by pharmacological stress.
